# Comparative Transcriptome Profiling Reveals Developmental Regulatory Networks in Rainbow Trout Eggs and Hatched Larvae

**DOI:** 10.3390/biology15201872

**Published:** 2026-10-16

**Authors:** Yanming Sui, Mijun Xu, Jingtao Liu, Yiyun Zhang, Yuanhao Ren, Bo Qin, Na Ying, Tingting Lin, Siping Li, Hanfeng Zheng, Lei Li

**Affiliations:** 1 Key Laboratory of East China Sea Fishery Resources Exploitation, Ministry of Agriculture and Rural Affairs, East China Sea Fisheries Research Institute, Chinese Academy of Fishery Sciences, Shanghai 200090, China; suiyanming@ycit.edu.cn (Y.S.); renyh@ecsf.ac.cn (Y.R.); dhsqb1026@126.com (B.Q.); xyz6622@126.com (N.Y.); lintt@ecsf.ac.cn (T.L.); lisiping@ecsf.ac.cn (S.L.); 2 Jiangsu (Marine) Salmon and Trout Science and Technology Innovation Center, Yellow Sea Laboratory for Blue Seed Industry, Yancheng 224000, China; 3 College of Marine and Biology Engineering, Yancheng Institute of Technology, No. 1, Middle Xiwang Avenue, Yancheng 224000, China; xumijun12138@outlook.com (M.X.); liujingtao0819@163.com (J.L.); zyy050106@126.com (Y.Z.)

**Keywords:** rainbow trout (*Oncorhynchus mykiss*), transcriptome sequencing, differentially expressed genes, embryonic development, hatched larvae, immune system, metabolic reprogramming, novel transcripts

## Abstract

Rainbow trout (*Oncorhynchus mykiss*) is a globally important cold-water aquaculture species, yet the molecular events underlying the transition from embryo to free-living larva remain poorly resolved. In this study, eukaryotic reference-based transcriptome sequencing was used to compare genome-wide gene expression profiles between eggs (eyed-egg stage) and newly hatched larvae of *O. mykiss*. A total of 618 differentially expressed genes (DEGs) were identified, of which 297 were upregulated and 321 downregulated. Gene Ontology (GO) enrichment analysis showed that DEGs were significantly enriched in immune system process, response to stimulus, and biological regulation. Within the immune system process category, 4 DEGs were upregulated and 11 were downregulated in larvae, indicating a more complex remodelling of immune-related gene expression than simple activation. Kyoto Encyclopedia of Genes and Genomes (KEGG) enrichment analysis identified only Glycolysis/Gluconeogenesis (map00010) as significantly enriched (padj < 0.05). Nitrogen metabolism (map00910) and tryptophan metabolism (map00380) showed enrichment trends but were not statistically significant after multiple-testing correction. Because these pathways contained only a small number of DEGs and displayed mixed regulation, no overall pathway-level up- or down-regulation was inferred. These results suggest a possible metabolic remodelling during the transition from yolk-dependent anabolism to catabolic utilization in larvae. Transcript assembly further identified 134,770 potential novel isoforms and 2902 intergenic transcripts classified as putative novel transcripts, enriching the genome annotation of *O. mykiss*. Trend clustering indicated coordinated regulation of immune and stress-response genes together with downregulation of amino acid and energy metabolism genes, revealing programmed temporal regulation during this developmental transition. These findings provide foundational data for understanding early development in rainbow trout and candidate molecular targets for health-oriented aquaculture management.

## Simple Summary

Understanding how rainbow trout develop from eggs to newly hatched larvae is important for improving fish farming and conservation. Here, we compared the genetic activity in unhatched eggs and in larvae that had just hatched, to identify which biological processes change most dramatically during this critical transition. The results showed that hundreds of genes are turned on or off as the embryo becomes a free-living larva. Genes involved in fighting infections and responding to environmental challenges undergo marked remodelling after hatching, with some being activated and others suppressed, which makes sense because the larva must now defend itself in the surrounding water. At the same time, genes responsible for building and storing nutrients are turned down, reflecting that the larva is shifting from using stored yolk to consuming its own energy reserves. The study also uncovered many putative novel transcripts, suggesting that rainbow trout have more developmental instructions than were previously recognized. These findings help explain how young fish adapt to life outside the egg and may guide fish farmers in creating better rearing conditions, ultimately supporting healthier and more sustainable aquaculture practices.

## 1. Introduction

Fish embryonic development represents a critical life stage that determines individual survival and successful population recruitment, and its orderly progression relies on a series of highly conserved and temporally coupled cellular and molecular events, including zygotic genome activation, germ layer differentiation, tissue morphogenesis, and organ primordium formation. Studies in the model species zebrafish (*Danio rerio*) have fully demonstrated that the precise temporal transition between maternal factors and the zygotic genome is a prerequisite for normal embryogenesis, and that dysregulation of any key node in this process may lead to developmental arrest or phenotypic abnormalities [1]. Research using this model has also revealed that metabolic microenvironments and small-molecule metabolites tightly regulate hatching and larval growth, highlighting evolutionary conservation of metabolic control during early ontogeny [2]. Collectively, these findings indicate that embryonic development is governed by sophisticated multi-layer molecular regulatory networks. Nevertheless, such detailed molecular knowledge remains limited for rainbow trout, a commercially valuable salmonid species.

In recent years, with the rapid development of high-throughput sequencing technologies, transcriptomics has become a core approach for elucidating the molecular basis of embryonic development in non-model fish species, with research foci expanding from traditional morphological observations to deeper levels including gene expression dynamics, epigenetic modifications, and signalling pathway regulation. By comparing transcriptomic profiles across different developmental stages, researchers can systematically identify key genes involved in specific developmental events and their co-regulated networks, without being constrained by prior hypotheses about candidate genes. For instance, multi-omics integrative studies in turbot (*Scophthalmus maximus*) have revealed that over 90% of active genes exhibit significant temporal differential expression during embryogenesis, and that dynamic remodelling of chromatin accessibility is closely associated with somitogenesis and early organogenesis, providing a comprehensive framework for understanding vertebrate development [3,4,5]. Similarly, integrated transcriptomic and metabolomic analyses have demonstrated unique advantages in elucidating the regulatory mechanisms of growth in various economically important fish species such as silver pomfret (*Pampus argenteus*) [6,7,8] and large-scale loach (*Paramisgurnus dabryanus*) [9,10], providing new perspectives for understanding the coupling between early development and growth regulation at the molecular level. Comparative transcriptomic approaches have also been successfully applied to investigate developmental transitions in other fish species, including the identification of stage-specific gene expression signatures associated with hatching and larval onset. These findings collectively demonstrate that transcriptome sequencing technology can effectively overcome the limitations of traditional morphological markers and candidate-gene approaches, comprehensively revealing the transcriptional regulatory landscape of embryonic development without prior assumptions, and offering a novel cognitive dimension for dissecting the early developmental mechanisms of economically important fish.

Rainbow trout (*Oncorhynchus mykiss*), a member of the family Salmonidae within the order Salmoniformes, is one of the most widely cultivated cold-water fish species globally, characterized by high economic value, substantial aquaculture production, and broad consumer acceptance. *O. mykiss* also represents a focal species of considerable research interest in evolutionary biology, comparative physiology, population genetics, and aquaculture breeding programmes, owing to its well-characterized biology and the availability of a high-quality reference genome assembly. The embryonic development of *O. mykiss* exhibits distinct stage-specific characteristics that are closely adapted to its natural cold-water habitat: fertilized eggs undergo a prolonged incubation period; a general range of 30 to 60 days has been reported in the literature under low-temperature conditions (typically 8–12 °C). Although the optimal developmental temperature for rainbow trout commonly ranges from 8 to 12 °C, we applied 16 °C in this experiment to accelerate embryonic development and shorten the experimental cycle under controlled laboratory conditions. Embryonic survival status was monitored throughout incubation, and no obvious abnormal mortality or morphological defects were observed under this temperature regime. In the present study, under a constant incubation temperature of 16 °C, hatching occurred at approximately 24 days post-fertilization (dpf), corresponding to approximately 384 degree-days. This transition from embryo (egg) to newly hatched larva is of particular biological significance, as it involves a fundamental shift in energy metabolism—from the catabolism of yolk-derived nutrients to the active foraging and digestion of external food sources—along with progressive functional maturation of the digestive, sensory, and locomotor systems. Moreover, this period represents the first exposure of the organism to external environmental signals and constitutes a critical window of survival rate fluctuation throughout the entire life cycle. To date, a series of molecular-level investigations have been conducted on *O. mykiss* embryogenesis, encompassing hormonal regulation of cyclic ovarian development [11], early gene expression differences between distinct ecotypic populations [12], and coordinated translational and transcriptional responses under thermal stress conditions [13,14,15].

Previous transcriptomic investigations in salmonids have largely concentrated on stress tolerance, aquaculture performance traits and pathogen responses in juvenile and adult individuals, with only limited attention to genome-wide transcriptional dynamics during early embryonic and larval development. The critical developmental transition from fertilized egg to newly hatched larva involves drastic physiological and morphological remodelling, yet the associated transcriptomic reprogramming, particularly the coordinated regulation of immune activation and metabolic remodelling, has not been systematically characterized in rainbow trout (*Oncorhynchus mykiss*).

Despite the availability of certain transcriptomic resources derived from various developmental stages or tissues, direct comparisons between the egg and the newly hatched larva in *O. mykiss* remain limited, and the molecular events and regulatory networks underpinning this transition have not been systematically characterized.

In salmonids, transcriptomic studies have begun to resolve stage-specific gene expression during early ontogeny. For example, studies in rainbow trout [16] and Atlantic salmon have characterized transcriptional responses to thermal stress and hypoxia during embryogenesis, and comparative analyses in other teleosts, such as the common perch, have identified hatching-associated immune and metabolic signatures in newly hatched larvae [17]. Hatching constitutes a critical developmental switch for teleost embryos, and transcriptome sequencing enables us to decode its underlying molecular events.

Despite growing transcriptomic resources for salmonids, direct comparisons between the egg and the newly hatched larva within a single species remain scarce, and the regulatory networks coordinating immune maturation and metabolic reprogramming across this transition are largely unresolved. We therefore tested two hypotheses: (1) that hatching is accompanied by remodelling of endogenous immune-related gene expression, reflecting the transition from maternal protection to autonomous defence; and (2) that this transition involves a metabolic shift from yolk-dependent anabolic storage to catabolic utilization. By comparing genome-wide expression profiles between eyed eggs and newly hatched larvae of *O. mykiss*, this study aimed to identify stage-specific DEGs, enriched pathways, and novel transcripts, thereby providing a transcriptomic framework for early development in this species.

To systematically elucidate the molecular regulatory features characterizing the transition from embryo to newly hatched larva in *O. mykiss*, this study employed eukaryotic reference-based transcriptome sequencing [18] to comprehensively compare genome-wide gene expression profiles between *O. mykiss* eggs and newly hatched larvae. The objective was to identify differentially expressed genes and their significantly enriched metabolic pathways, thereby revealing the biological processes and regulatory networks involved in this critical developmental transition. The findings are expected to provide foundational data for understanding the molecular basis of early development in *O. mykiss*, to extend the current transcriptomic resources available for salmonid developmental biology, and to offer potential molecular targets and theoretical references for optimizing seedling rearing conditions, improving hatchery practices, and formulating health-oriented aquaculture management strategies for this commercially valuable species.

## 2. Materials and Methods

### 2.1. Experimental Materials and Triploid Induction

Broodstock of *O. mykiss* were reared at the aquaculture base of the East China Sea Fisheries Research Institute, Chinese Academy of Fishery Sciences. Artificial fertilization was performed following standard dry fertilization procedures [19]. Triploid induction was performed with minor modifications to the published method. Briefly, at 20–25 min post-fertilization (at water temperature of 16 °C, prior to the extrusion of the second polar body), fertilized eggs of *O. mykiss* were transferred to a hydrostatic pressure apparatus and subjected to a pressure of 65 MPa for 6–8 min to inhibit second polar body release, thereby retaining one set of chromosomes from the second polar body and generating triploid zygotes. The treated eggs were incubated in a recirculating freshwater system at a constant temperature of 16 °C. Under this condition, hatching occurred at approximately 24 days post-fertilization (dpf). To verify triploidy induction rate, 30 individuals were randomly selected after hatching, and fin clips were collected. DNA relative content was measured using a flow cytometer (CyFlow^®^ Ploidy Analyzer, Partec, Görlitz, Germany), with known diploid *O. mykiss* as controls, to confirm ploidy [20] status and induction success rate. All experimental procedures were approved by the Institutional Animal Care and Use Committee of the East China Sea Fisheries Research Institute and were performed in strict accordance with the principles of laboratory animal welfare and ethics. Triploid *O. mykiss* were used in this study because triploidy is of commercial interest in salmonid aquaculture, offering advantages such as sterility and improved growth performance. However, triploidy is also known to impose genomic stress that can affect gene expression and development. The results presented here should therefore be interpreted in the context of triploid physiology, and extrapolation to diploid *O. mykiss* requires caution.

### 2.2. Sample Collection and Incubation Conditions

Fertilized eggs of *O. mykiss* were incubated in a recirculating freshwater system at a constant water temperature of 16 °C maintained by a chiller [21]. The incubation water consisted of aerated freshwater with dissolved oxygen maintained at 8–9 mg/L, and the incubation environment was maintained under weak-light or dark conditions [22]. Egg quality was inspected daily, and dead eggs (opaque or mouldy) were promptly removed, followed by disinfection treatment to prevent water contamination and cross-infection.

Two developmental stages were selected for sampling: Group A comprised eggs at approximately 24 days post-fertilization (dpf; eyed-egg stage, unhatched embryos; ~384 degree-days). Group B comprised newly hatched larvae at 31 dpf (corresponding to approximately 7 dph; ~496 degree-days). The interval between the two sampling points was therefore approximately 7 days (~112 degree-days). Three biological replicates were collected for each group, designated as A_1, A_2, A_3 and B_1, B_2, B_3, respectively. All samples were immediately snap-frozen in liquid nitrogen and subsequently stored at −80 °C until RNA extraction.

The two sampling points were selected based on the following considerations. The eyed-egg stage (24 dpf) represents a critical embryonic milestone at which the optic vesicles are fully formed, the embryo is morphologically recognizable, and major organ primordia have been established. The larval stage at 7 dph represents the early free-living phase, during which the digestive, respiratory, and immune systems undergo rapid functional maturation, and the organism is transitioning from yolk-dependent anabolism to catabolic utilization. Together, these two stages bracket the developmental transition from a protected embryonic existence to a free-living larval existence, which is the central focus of this study.

No exogenous feed was provided throughout the experiment. At 31 dpf (approximately 7 dph), all sampled larvae still possessed a visible residual yolk sac, confirming that they remained exclusively dependent on endogenous yolk reserves. Under our incubation conditions (16 °C), first exogenous feeding in O. mykiss is expected at approximately 10–14 days post-hatching (~160–224 degree-days) under 16 °C [23], which is considerably later than the 7 dph sampling point used here.

### 2.3. RNA Extraction and Quality Assessment

Total RNA was extracted from each sample using TRIzol^®^ reagent (Invitrogen, Carlsbad, CA, USA). RNA quality was assessed using the following methods: (1) RNA concentration and purity were measured using a Nanodrop 2000 spectrophotometer (Thermo Scientific, Rockford, IL, USA), with OD260/280 ratios recorded; (2) RNA integrity was evaluated by 1% agarose gel electrophoresis; and (3) the RNA integrity number (RIN) was determined using an Agilent 5300 Bioanalyzer (Agilent Technologies, Santa Clara, CA, USA). RNA samples meeting all of the following criteria [24] were used for subsequent library construction: total RNA ≥ 1 μg, concentration ≥ 30 ng/μL, RIN > 6.5, and OD260/280 ratio between 1.8 and 2.2.

### 2.4. cDNA Library Construction and Sequencing

From each qualified total RNA sample, mRNA with poly(A) tails was enriched using Oligo(dT)-conjugated magnetic beads via A-T bases (Dynabeads® Oligo(dT)_25_, Thermo Fisher Scientific, Carlsbad, CA, USA) via A-T base pairing. The enriched mRNA was then fragmented into approximately 300 bp short fragments by addition of fragmentation buffer under optimized reaction conditions. Using the fragmented mRNA as template, first-strand cDNA was synthesized with random primers and reverse transcriptase, followed by second-strand cDNA synthesis to generate stable double-stranded cDNA. The double-stranded cDNA was subjected to end-repair to convert sticky ends into blunt ends, and an “A” base was added to the 3′ ends, followed by adapter ligation. After purification, the adapter-ligated products were size-selected using magnetic beads, and the target fragments were enriched by PCR amplification to obtain the final sequencing library.

After library construction, precise quantification was performed using a Qubit^®^ 4.0 fluorometer (Invitrogen, Carlsbad, CA, USA). Libraries were pooled in proportion to the expected data output and sequenced on the NovaSeq X Plus [25] platform (Illumina, San Diego, CA, USA) with a paired-end strategy and a read length of 150 bp (PE150).

### 2.5. Sequencing Data Quality Control and Filtering

Raw image signals generated from sequencing were converted into raw sequencing reads (raw reads) through base calling using the platform-supplied software and stored in FASTQ format. Quality control and filtering of raw data were performed using fastp (v0.23.4) [26], with the following steps: (1) removal of reads containing adapter sequences; (2) removal of reads with an excessively high proportion of ambiguous bases (N bases); (3) removal of reads with a high proportion of low-quality bases (quality score Q < 20); and (4) removal of reads that became too short after filtering. The high-quality clean reads obtained after the above quality control steps were used for all subsequent analyses.

### 2.6. Sequence Alignment

The *O. mykiss* reference genome obtained from the NCBI database (GenBank accession numbers: NC_048565.1–NC_048574.1) was used as the alignment reference. Clean reads from each sample were aligned to the reference genome using HISAT2 (v2.2.1) [27] with default parameters. After alignment, the total mapping rate, uniquely mapped rate, and multi-mapped rate were calculated for each sample to evaluate alignment quality.

### 2.7. Transcriptome Assembly and Novel Transcript Prediction

Based on the alignment results for each sample, transcriptome assembly and reconstruction were performed using StringTie (v2.2.1) [28]. The assembled transcripts were compared with the reference genome annotations, and potential novel transcripts were identified based on the Class code classification system [29]. Specifically, Class code “u” (unknown intergenic transcripts) were defined as putative novel transcripts, while Class codes “x” (exonic overlap with reference on the opposite strand), “i” (transfrags falling entirely within a reference intron), and “j” (potentially novel isoforms sharing at least one splice junction with the reference transcript) were all categorized as potential novel transcripts.

### 2.8. Functional Annotation

The sequences of all coding genes, including known genes and predicted novel genes, were aligned against the following public databases for functional annotation: NR (NCBI non-redundant protein database), Swiss-Prot, GO (Gene Ontology) [30], KEGG [31] (Kyoto Encyclopedia of Genes and Genomes), EggNOG, and Pfam. Sequence alignments were performed using DIAMOND (v0.9.24) [32] in BLASTP (BLAST+ v2.13.0) mode with a significance threshold of E-value ≤ 1 × 10^−5^. The annotation results were integrated by consolidating the best matching information from each database to obtain functional descriptions, GO classifications, and KEGG pathway assignments for each gene, and the number and proportion of annotated genes in each database were summarized.

### 2.9. Gene Expression Quantification

Gene expression levels were quantified using RSEM (v1.3.3) [33]. Based on the alignment results and the reference genome annotation file for each sample, the raw read counts for each gene were calculated. The read counts were then converted to TPM (Transcripts Per Million) [34] values as normalized gene expression estimates for all subsequent expression analyses.

### 2.10. Sample Relationship Analysis

To evaluate the consistency among biological replicates and the differences between groups, the following analyses were performed:

(1) Venn analysis: The actively expressed gene sets (mean TPM ≥ 1 within each group) in Group A and Group B were compared, and a Venn diagram was generated to display the numbers of genes shared between or unique to each group. A total of 25,955 actively expressed genes were included in this analysis.
(2) Correlation analysis: Based on TPM values of all genes in each sample, Pearson correlation coefficients between samples were calculated, and hierarchical clustering was applied to cluster the samples.
(3) Principal component analysis (PCA): PCA was performed using the expression matrix of all genes across samples with the prcomp function in R software (v4.2.1) [35]. PCA scatter plots were generated with PC1 and PC2 as axes to evaluate the similarity of expression profiles among samples.

### 2.11. Differential Expression Analysis

Differential expression analysis was performed using DESeq2 (v1.42.0) [36], with gene-level raw read counts as input and a negative binomial distribution model for significance testing. The comparison was set as Group B versus Group A (with Group A as the control). Differentially expressed genes (DEGs) were identified using the following thresholds: |log_2_(fold change)| ≥ 1 and Benjamini–Hochberg-adjusted [37] *p*-value (padjust) < 0.05. The direction of regulation was determined based on the sign of log_2_(fold change): log_2_(FC) > 0 indicated up-regulation, and log_2_(FC) < 0 indicated down-regulation. Results were visualized using volcano plots and MA plots.

### 2.12. Functional Enrichment Analysis

GO enrichment analysis: GO enrichment analysis of DEGs was performed using GOATOOLS (v0.6.5) [38]. Fisher’s exact test was used to calculate the enrichment significance of each GO term among the DEGs, with all annotated genes serving as the background set. Multiple testing correction was applied using the Benjamini–Hochberg method, and padjust < 0.05 was considered statistically significant. Results were summarized into three main categories: Biological Process (BP), Cellular Component (CC), and Molecular Function (MF), and visualized using bar plots and bubble plots.

KEGG pathway enrichment analysis: KEGG pathway enrichment analysis of DEGs was conducted using R scripts implementing the KOBAS (v2.1.1) [39] algorithm. The computational approach was identical to that used for GO enrichment analysis, and padjust < 0.05 was set as the threshold for significantly enriched pathways. Significantly enriched KEGG pathways were further categorized into seven major classes (Metabolism, Genetic Information Processing, Environmental Information Processing, Cellular Processes, Organismal Systems, Human Diseases, and Drug Development). In addition, Gene Set Enrichment Analysis (GSEA, v4.3.2) was performed to detect coordinated transcriptional changes across the entire ranked transcriptome, without relying on arbitrary DEG cutoff thresholds. The GO terms and KEGG pathways obtained from the above functional annotation were used as predefined gene sets. All genes were ranked according to the signal-to-noise metric between the larval group (Group B) and the eyed-egg group (Group A). A total of 1000 phenotype permutations were conducted, and a false discovery rate (FDR) < 0.05 was set as the significance threshold for enrichment.

### 2.13. Clustering Analysis

Hierarchical clustering analysis was performed on the TPM expression matrix of DEGs using Euclidean distance as the distance metric and complete-linkage method for clustering. The clustering results were displayed as a heatmap, with red and blue representing high and low expression levels, respectively. Based on the expression trends, genes with similar expression patterns were grouped into distinct clusters, and expression trend plots were generated for each cluster.

## 3. Results

### 3.1. Sequencing Data Quality Control and Alignment to the Reference Genome

To investigate transcriptomic differences between eyed eggs and newly hatched larvae of *O. mykiss*, we performed RNA-seq across six samples comprising three biological replicates per developmental stage. After quality filtering, all samples attained Q30 values above 92%, with low sequencing error rates, confirming high-quality sequencing data suitable for downstream analyses. Read mapping against the *O. mykiss* reference genome achieved overall mapping rates ranging from 94.97% to 95.57%, demonstrating minimal exogenous contamination.

The clean reads were aligned to the *O. mykiss* reference genome (NCBI assembly) using HISAT2. Alignment statistics are summarized in Table 1. Total mapping rates ranged from 94.97% to 95.57%, uniquely mapped reads accounted for 87.25–87.66%, and multi-mapped reads for 7.48–8.31%. The high proportions of uniquely mapped reads confirmed the absence of exogenous contamination and validated the choice of reference genome, providing a solid foundation for transcript assembly and quantification.

### 3.2. Transcript Assembly and Novel Transcript Prediction

The mapped reads were assembled using StringTie and compared with the reference annotation. Most assembled transcripts contained 1–10 exons, and most genes had 1–3 transcript isoforms, consistent with the structural features of eukaryotic transcripts.

Novel transcript prediction (Table 2) identified 134,770 potential novel isoforms (class code “j”), of which 2902 were intergenic transcripts (class code “u”) defined as putative novel transcripts. In addition, 134,606 transcripts were complete matches to known transcripts (“=”), and 1170 had exonic overlap with reference transcripts (“o”). These results indicate previously unannotated transcriptional events during *O. mykiss* development.

### 3.3. Functional Annotation Overview

To comprehensively assess gene functions, all 44,665 genes with detectable transcription signals across all samples were annotated against six databases: NR, SwissProt, GO, KEGG, EggNOG, and Pfam. Among them, 25,955 genes met the criterion of mean TPM ≥ 1 within groups and were defined as actively expressed genes for subsequent sample relationship analysis.

Genes and transcripts expressed in samples refer to those with detectable expression signals in the sequencing samples of this study. Reference-annotated genes and transcripts refer to those annotated in the *O. mykiss* reference genome assembly. Table 3 counts include all genes with detectable transcription signals across all samples (total 44,665), not limited to actively expressed genes filtered by expression abundance.

### 3.4. Expression Distribution and Sample Relationships

**Figure 1:**
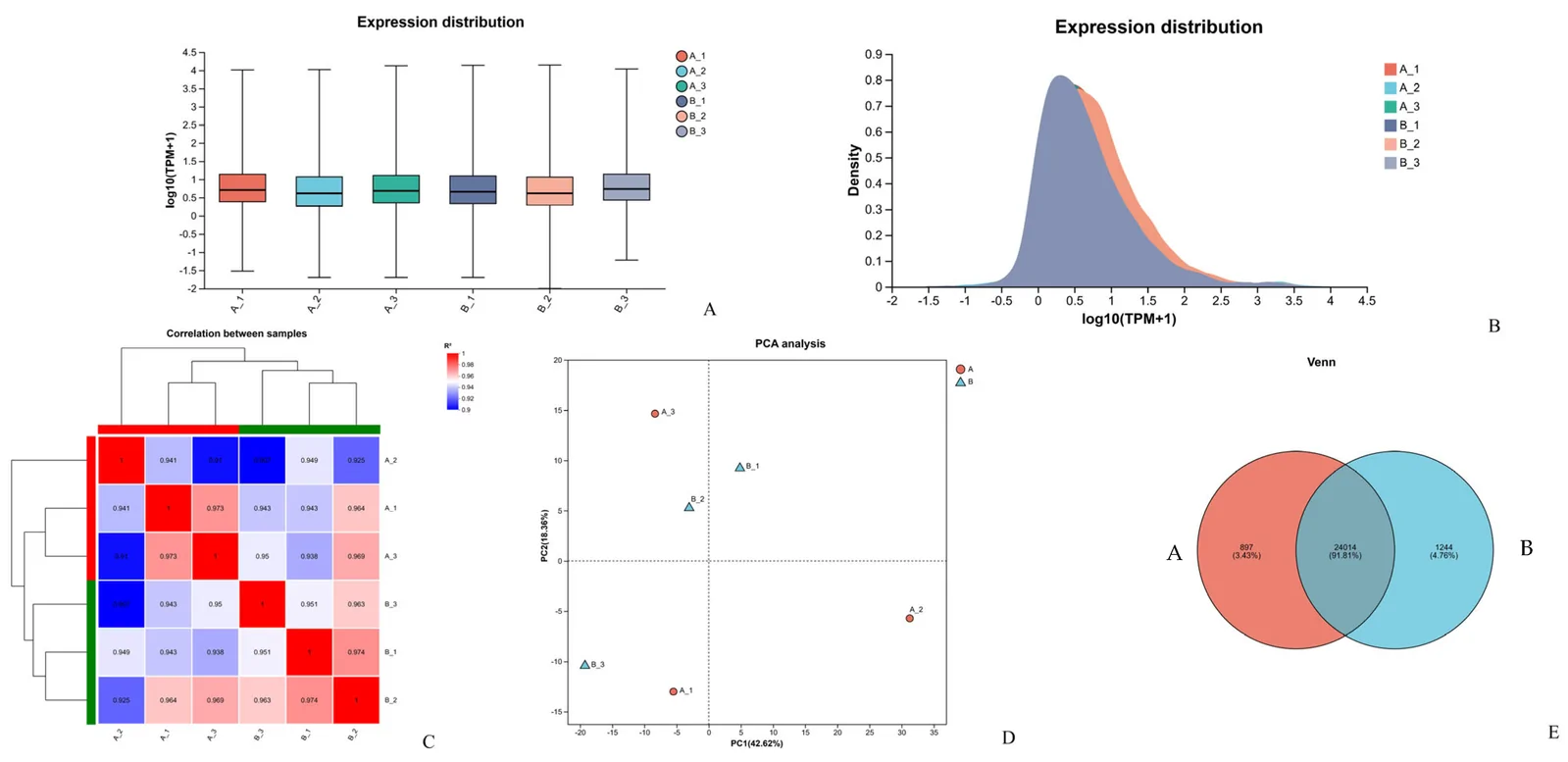
Expression distribution and sample-relationship analysis of *Oncorhynchus mykiss* eyed-egg group A (*n* = 3 biological replicates) and newly hatched larval group B (*n* = 3 biological replicates). (**A**) Boxplot of \​(\​log_{10}(\text{TPM} + 1)\​) values for all expressed genes per sample; horizontal central line = median, box = interquartile range. (**B**) Density plot showing \​(\​log_{10}(\text{TPM} + 1)\​) expression-value distribution. (**C**) Pearson-correlation heatmap across six samples based on TPM of all expressed genes; hierarchical clustering was implemented with Euclidean distance and complete-linkage method in R. (**D**) Principal-component analysis (PCA) performed in R prcomp function; PC1 and PC2 explained 42.62% and 18.36% of total variance, respectively (cumulative 60.98%). (**E**) Venn diagram displaying overlap of actively expressed gene sets (mean TPM ≥ 1). Numbers in parentheses represent percentage relative to total actively expressed genes. TPM, transcripts per million.

Based on TPM values, boxplots (Figure 1A) showed similar median expression across samples, though group B (larvae) exhibited slightly greater dispersion than group A (eggs). Density curves (Figure 1B) indicated broadly similar distributions, but with a slightly higher density at low expression levels in group B, suggesting downregulation of certain genes in larvae. Sample correlation analysis (Figure 1C) showed that intra-group Pearson correlation coefficients were all above 0.95, indicating good reproducibility; inter-group correlations were slightly lower but still high, suggesting overall similarity with detectable differences. Principal component analysis (PCA, Figure 1D) further confirmed that PC1 and PC2 together explained >70% of the variance, and the two groups were clearly separated, demonstrating distinguishable transcriptomic profiles between eggs and larvae. The Venn diagram (Figure 1E) illustrated shared and unique gene counts, further supporting inter-group differences.

### 3.5. Identification of Differentially Expressed Genes

**Figure 2:**
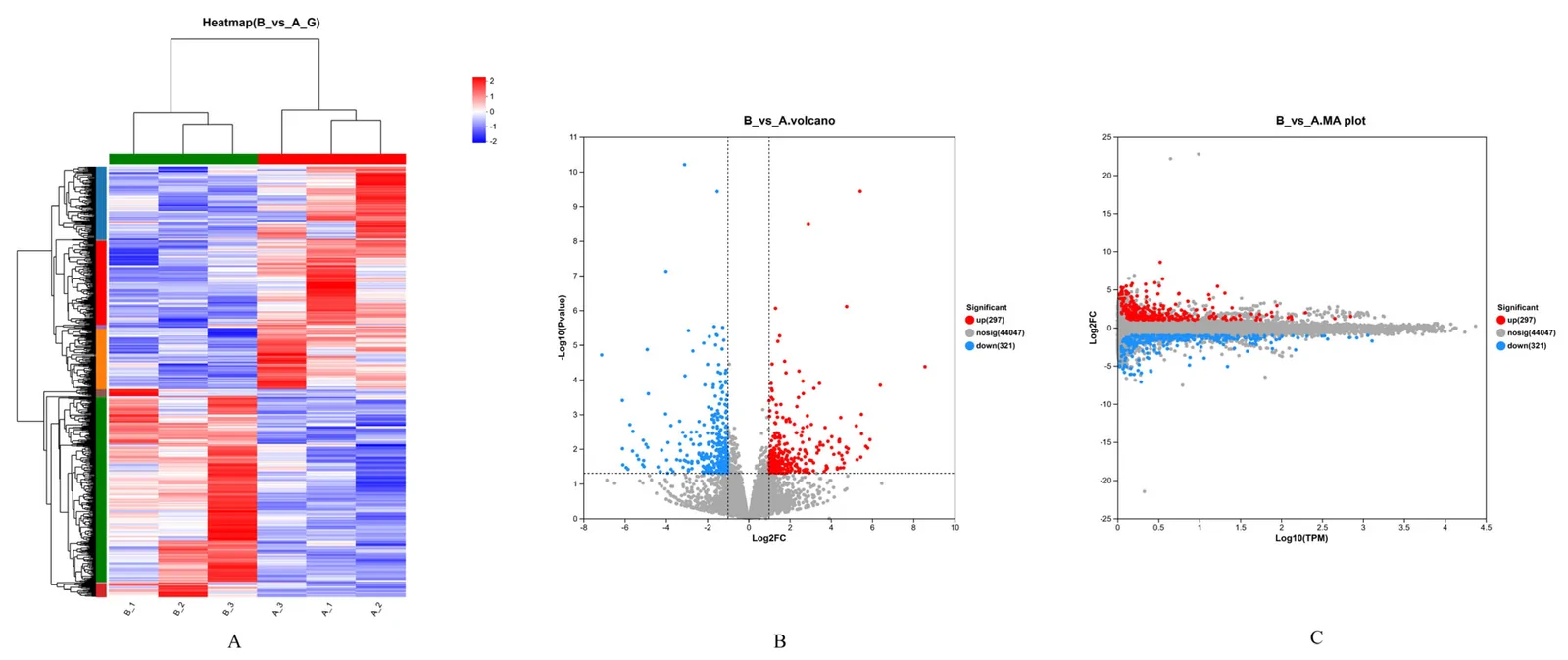
Differential-expression analysis comparing larval group B against eyed-egg group A (*n* = 3 biological replicates per group). (**A**) Hierarchical-clustering heatmap for 618 differentially expressed genes (DEGs). Each row corresponds to one DEG; each column is one biological sample. Expression matrix was Z-score normalized across samples before clustering. Red = high relative expression; blue = low relative expression. Clustering: Euclidean distance, complete-linkage method. (**B**) Volcano plot of gene-expression changes. DEG cutoff thresholds: \​(|\log_2(\text{fold change})|\ge 1\​), Benjamini–Hochberg adjusted *p*-value (padjust) < 0.05. Red dots: significantly up-regulated DEGs (297); blue dots: significantly down-regulated DEGs (321); grey dots: non-DEGs. (**C**) MA plot showing mean gene-expression abundance versus \​(\log_2(\text{fold change})\​). Thresholds and colour scheme are identical to panel (**B**). DEG, differentially expressed gene.

Differential expression analysis between group B (larvae) and group A (eggs) was performed using DESeq2 with thresholds of |log_2_FC| ≥ 1 and padjust < 0.05. A total of 618 DEGs were identified, with 297 upregulated and 321 downregulated, as summarized in Table 4. Representative expression differences are shown in Figure 2 (volcano plot and heatmap).

### 3.6. GO Functional Enrichment Analysis of DEGs

**Figure 3:**
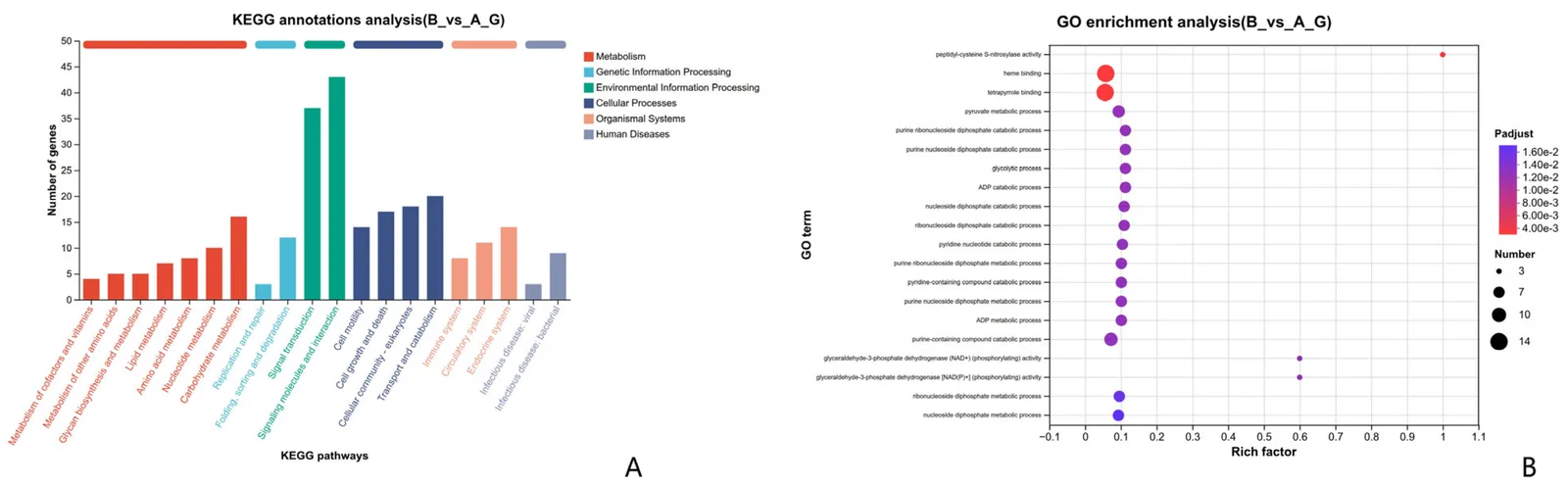
GO functional-enrichment analysis for DEGs (comparison B vs. A; *n* = 3 biological replicates per group). (**A**) GO-classification bar chart presenting counts of DEGs assigned to three main Gene-Ontology categories: Biological Process, Cellular Component, Molecular Function. (**B**) Bubble plot for top enriched GO terms. *X*-axis: rich factor; *Y*-axis: GO-term label; bubble size: number of DEGs mapped to each GO term; colour gradient: Benjamini–Hochberg adjusted *p*-value (padjust). Enrichment was calculated using GOATOOLS with Fisher’s exact test; significance threshold padjust < 0.05. DEG, differentially expressed gene; GO, Gene Ontology.

To explore the biological functions of DEGs, GO enrichment analysis was performed using GOATOOLS (*padj* < 0.05). Enriched terms were classified into biological process (BP), cellular component (CC), and molecular function (MF), as shown in Figure 3.

In the BP category, DEGs were significantly enriched in “immune system process” (GO:0002376), “biological regulation” (GO:0065007), “homeostatic process” (GO:0042592), “reproductive process” (GO:0022414), “cellular process” (GO:0009987), “developmental process” (GO:0032502), “multicellular organismal process” (GO:0032501), “localization” (GO:0051179), and “response to stimulus” (GO:0050896) (Table 5), indicating that immune activation and stress adaptation are key events during development. In the CC category, terms such as “membrane-bounded organelle” and “cytoskeleton” were enriched, suggesting cellular structural remodelling. In the MF category, “protein binding” and “signalling receptor binding” were prominent, indicating dynamic regulation of protein interaction networks (full lists are provided in Supplementary Materials). The bar chart (Figure 3A) and bubble plot (Figure 3B) visualized the enrichment, revealing profound reprogramming of immune defence, stress perception, and intracellular organization during the transition from eggs to larvae.

### 3.7. KEGG Pathway Enrichment Analysis of DEGs

**Figure 4:**
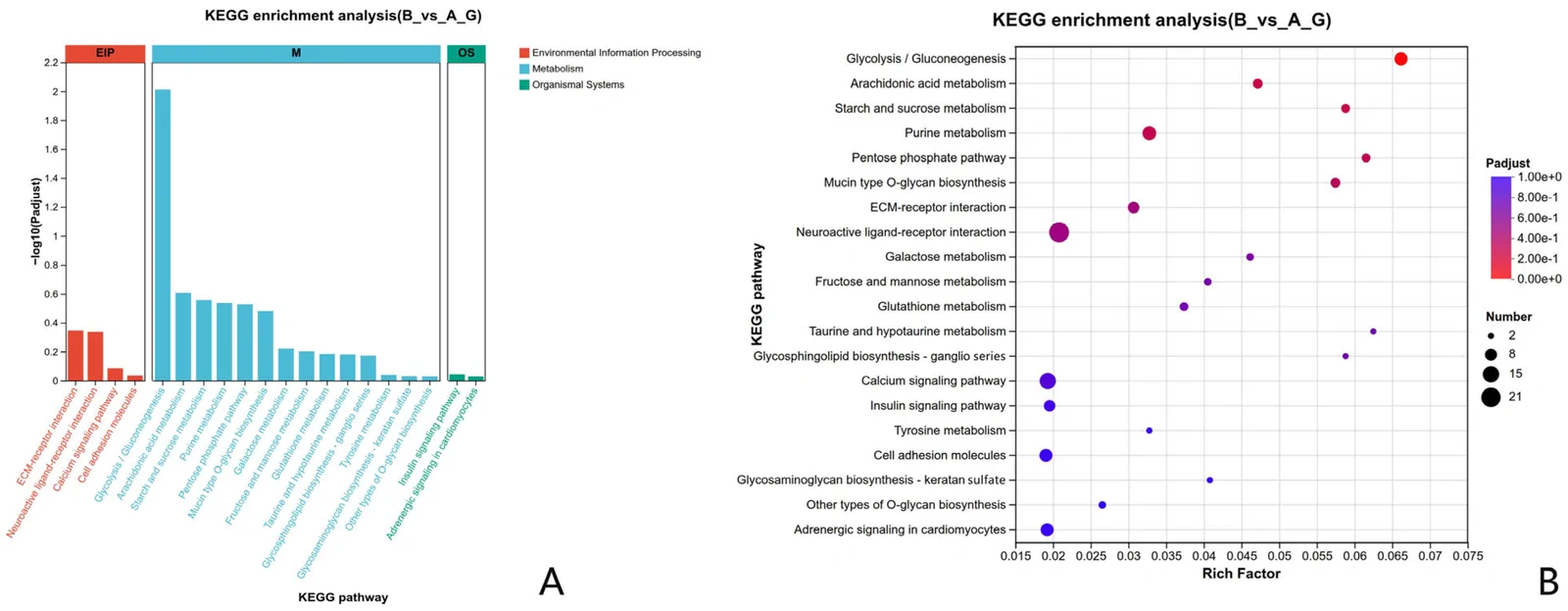
KEGG pathway-enrichment analysis for DEGs (comparison B vs. A; *n* = 3 biological replicates per group). (**A**) KEGG-classification bar chart showing counts of DEGs annotated to major KEGG functional categories. (**B**) Bubble plot of enriched KEGG pathways. *X*-axis: rich factor; *Y*-axis: pathway name; bubble size: number of DEGs within the pathway; colour gradient: Benjamini–Hochberg adjusted *p*-value (padjust). Enrichment analysis was implemented by KOBAS algorithm with significance threshold padjust < 0.05. DEG, differentially expressed gene; KEGG, Kyoto Encyclopedia of Genes and Genomes.

KEGG pathway enrichment analysis of DEGs revealed that, among 218 mapped pathways, only Glycolysis/Gluconeogenesis (map00010) was significantly enriched (*padj* < 0.05). Nitrogen metabolism (map00910) and tryptophan metabolism (map00380) showed enrichment trends but did not reach statistical significance after multiple testing correction (Table 6). All three pathways belong to the “Metabolism” category. DEGs within these pathways presented a mixed regulation pattern with both upregulated and downregulated genes, and we did not infer overall pathway-level up- or down-regulation due to the limited number of DEGs per pathway. The KEGG classification bar chart (Figure 4A) and bubble plot (Figure 4B) illustrate the enrichment significance. These enriched metabolic pathways suggest that energy and nitrogen metabolism may be remodelled during the egg-to-larva transition.

#### Gene Set Enrichment Analysis (GSEA)

To complement the threshold-based enrichment analysis of DEGs and detect coordinated transcriptional changes at the whole-transcriptome level, we performed Gene Set Enrichment Analysis (GSEA) using GO terms and KEGG pathways as predefined gene sets. Genes were ranked by the signal-to-noise metric between Group B (larvae) and Group A (eggs), with 1000 permutations and a significance threshold of FDR < 0.05.

For GO gene sets, two distinct functional enrichment patterns were observed. Gene sets significantly enriched in Group A (eyed-egg stage, negative NES) were mainly associated with anabolic and metabolic processes, including gluconeogenesis, lipoprotein metabolism, hexose biosynthesis, hemoglobin complex formation, collagen trimer assembly, and reactive oxygen species metabolism, consistent with the active biosynthesis and nutrient storage status during embryonic development. Gene sets significantly enriched in Group B (larval stage, positive NES) were predominantly related to visual system development and neural function, such as structural constituent of eye lens, lens development, visual perception, postsynaptic membrane specialization, glutamatergic synaptic transmission, and nervous system development, reflecting the functional maturation of sensory and neural systems after hatching.

For KEGG pathways, nine metabolic pathways were significantly enriched in Group A (FDR < 0.05, negative NES), including glycolysis/gluconeogenesis, starch and sucrose metabolism, pyruvate metabolism, pentose phosphate pathway, and porphyrin metabolism. These results were consistent with the metabolic features observed in DEG-based enrichment but provided stronger statistical support at the whole gene-set level. No KEGG pathways reached the significance threshold of FDR < 0.05 in Group B; only developmental and signalling-related pathways such as melanogenesis, adrenergic signalling in cardiomyocytes, and Wnt signalling pathway showed weak enrichment trends.

Full GSEA statistics, overview bubble plots and representative enrichment plots are provided in Supplementary Materials.

### 3.8. Clustering Analysis of DEGs

**Figure 5:**
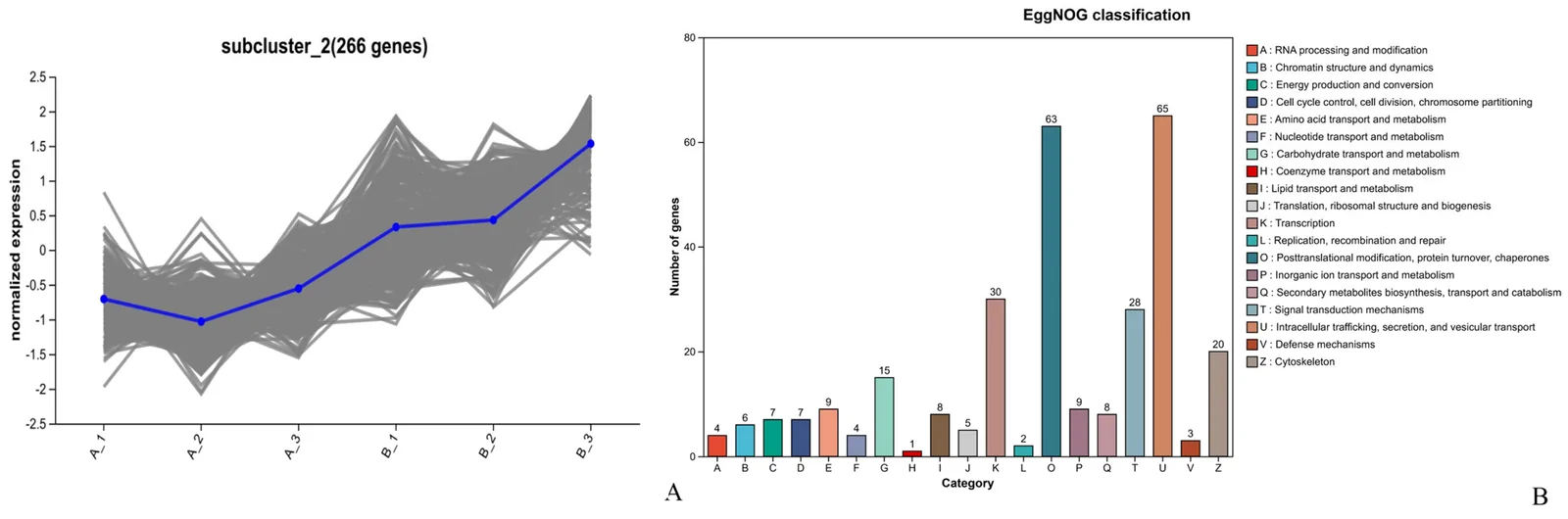
Trend-clustering analysis of total 618 DEGs between eyed-egg (group A) and newly hatched-larva (group B) stages (*n* = 3 biological replicates per group). A total of 10 expression subclusters were generated for all 618 DEGs. (**A**) Expression-trend plot for subcluster_2 containing 266 genes. Grey lines represent Z-score-normalized expression profiles for individual DEGs; solid blue line indicates the average expression trend for genes within this subcluster. Trend clustering was performed on Z-score-normalized TPM matrix of all DEGs. (**B**) EggNOG functional-classification bar chart for genes from subcluster_2, showing gene number for each EggNOG orthologous category. DEG, differentially expressed gene; TPM, transcripts per million.

To further resolve the dynamic expression patterns of the 618 DEGs during the egg-to-larva transition, trend clustering analysis was performed (Figure 5A). This analysis partitioned the DEGs into several distinct expression trend subsets. Trend-clustering classified all 618 DEGs into 10 distinct expression subclusters. Subcluster 2, illustrated in Figure 5, represents one major gene module showing increased expression from eyed egg to larval stage, reflecting a representative programmed temporal expression pattern during early rainbow trout development. These clustering results provide important clues for identifying core regulatory networks specific to developmental stages.

## 4. Discussion

In this study, transcriptome sequencing was employed to systematically compare the gene expression profiles of two consecutive developmental stages of *O. mykiss*—eggs and newly hatched larvae. A total of 618 differentially expressed genes were identified, of which 297 were upregulated and 321 downregulated. Combined with functional enrichment analysis and expression trend clustering, our findings reveal the transcriptomic reprogramming accompanying the transition from embryonic subsistence to free-living in *O. mykiss*, primarily manifested as the coordinated changes in immune system maturation, metabolic pathway switching, and transcriptional regulatory networks.

### 4.1. Developmental Stage Transition Is Accompanied by Remodelling of Immune-Related Gene Expression

In the present study, DEGs were significantly enriched in GO terms including “immune system process” (GO:0002376), “response to stimulus” (GO:0050896), and “biological regulation” (GO:0065007) during the transition from eggs to larvae, with 4 genes upregulated and 11 downregulated (Figure 3), indicating a coordinated remodelling of immune-related gene expression rather than simple unidirectional activation. This finding is highly consistent with the well-established pattern of progressive immune system maturation during early development in teleosts [40]. *O. mykiss* embryos primarily rely on maternally derived antibodies and non-specific immune factors within the egg chorion for protection during the embryonic stage [41]. Following hatching, larvae enter the external aquatic environment and face multiple stressors, including pathogenic microorganisms, temperature fluctuations, and osmotic challenges [42]. Therefore, the remodelling of immune-related gene expression is critical for larval survival. The complex regulation of immune-related genes observed in this study likely reflects an adaptive response initiated by larvae to establish autonomous immune defence upon hatching. In addition, multiple genes within the “response to stimulus” category were also upregulated, further supporting the enhanced capacity for stress perception and response in the larval stage. Notably, 11 of the 15 immune system process DEGs were downregulated, while 4 were upregulated, suggesting that the establishment of the immune system is not a simple unidirectional activation but involves fine-tuned homeostatic regulation [43].

### 4.2. Enrichment of Metabolic Pathways During the Transition from Anabolic Storage to Catabolic Utilization

KEGG pathway enrichment analysis based on DEGs showed that Glycolysis/Gluconeogenesis (map00010) reached statistical significance among the set of DEGs. Notably, Gene-Set Enrichment Analysis (GSEA) across the full transcriptome returned a negative normalized enrichment score (NES) for this pathway, indicating higher overall pathway-level transcriptional activity in eyed-egg group A rather than in larvae. DEGs mapped to this pathway displayed mixed up- and down-regulation patterns; therefore, we cannot infer simple overall pathway activation within larvae. Nitrogen metabolism (map00910) and tryptophan metabolism (map00380) (Figure 4) showed enrichment trends without statistical significance after multiple-testing correction. All three belong to the “Metabolism” category. DEGs in these pathways exhibited a mixed regulatory pattern with both upregulation and downregulation; given the small number of DEGs per pathway, we did not infer the overall direction of regulation at the pathway level. All three belong to the “Metabolism” category. DEGs in these pathways exhibited a mixed regulatory pattern with both upregulation and downregulation; given the small number of DEGs per pathway, we did not infer the overall direction of regulation at the pathway level. These enrichment features are consistent with metabolic remodelling during early *O. mykiss* development.

Fish eggs are rich in yolk, which stores substantial amounts of proteins, lipids, and glycogen to meet the energy demands of embryonic development, and anabolic processes predominate during this stage [44]. After hatching, exogenous feeding is not yet fully established, and internal yolk reserves continue to be depleted, forcing a gradual shift toward catabolic utilization [45]. The enrichment of Glycolysis/Gluconeogenesis may reflect reorganization of carbohydrate metabolism and energy supply during this transition. The enrichment of nitrogen metabolism may be related to amino acid turnover and ammonia handling [46], although the small number of DEGs limits strong conclusions. Tryptophan metabolism is closely associated with serotonin synthesis and immune regulation [47]; however, given the limited number of DEGs and the absence of pathway-level direction inference, this pathway should also be interpreted cautiously.

Under our incubation conditions, no exogenous feed was provided. At 7 dph, all sampled larvae still possessed a visible residual yolk sac, confirming that they remained exclusively dependent on endogenous yolk reserves. First exogenous feeding in *O. mykiss* under these conditions typically occurs at approximately 10–14 days post-hatching, considerably later than the 7 dph sampling point. Thus, the metabolic pathway enrichment observed at 7 dph reflects the endogenous yolk-dependent stage rather than the nutritional transition associated with exogenous feeding. These metabolic shifts observed between the eyed-egg stage and post-hatching larva are primarily triggered by progressive consumption of yolk-sac reserves. As yolk nutrients are gradually catabolized, the larva remodels its energy-related metabolic pathways to prepare physiologically for the upcoming transition to exogenous feeding. This metabolic reprogramming is biologically critical for early-stage larvae. Insufficient metabolic adjustment may disturb energy homeostasis before first feeding, which can reduce larval survival capacity and impair their adaptation to external food sources once exogenous feeding commences.

Similar metabolic transitions have been reported in newly hatched larvae of other teleosts, including the common perch, suggesting that metabolic remodelling may be a conserved feature of early teleost development [17]. Collectively, the enrichment of these metabolic pathways supports the view that the transition from eggs to larvae may involve a shift from an anabolic mode dependent on maternally derived energy reserves to a catabolic mode in which the organism relies on its own metabolic processes to maintain energy supply. This transition may lay the metabolic foundation for the subsequent establishment of exogenous feeding behaviour.

Consistent with the morphological maturation of sensory systems during the hatching transition, our GSEA results revealed significant enrichment of gene sets associated with visual perception and neural development in newly hatched larvae. Notably, this transcriptional signature does not contradict our core findings of metabolic reprogramming and immune activation. Instead, these biological events are coordinated, parallel developmental programmes triggered upon hatching. The metabolic switch from yolk-dependent nutrition to exogenous feeding supplies energy to support rapid neural and sensory tissue maturation, while immune pathway activation primes larvae to cope with pathogenic threats in the external aquatic environment. Collectively, the egg-to-larva transition represents a multifaceted developmental shift that integrates metabolic remodelling, immune priming, and functional establishment of neural and sensory systems; all these processes are critical prerequisites for larval survival after hatching.

### 4.3. Transcriptional Regulatory Networks and the Potential Functions of Novel Transcripts

Through transcript assembly and novel transcript prediction, we identified 134,770 potential novel isoforms and 2902 intergenic transcripts classified as putative novel transcripts. This result suggests that the *O. mykiss* genome may harbour a large number of previously unannotated transcriptional units that play regulatory roles during early development [48]. Notably, the number of novel transcripts far exceeded that of known transcripts, which may be attributable to the incomplete annotation of the *O. mykiss* genome, but also likely reflects the widespread presence of developmentally specific transcripts. In teleosts, early development is often the stage with the highest transcriptomic diversity, during which numerous non-coding RNAs and alternative splicing events are activated to participate in the fine-tuned regulation of cell differentiation and tissue morphogenesis [49]. The novel transcripts identified in this study, particularly the intergenic transcripts, may include long non-coding RNAs, which are known to play important roles in regulating the expression of genes involved in embryonic development [50]. However, as functional validation of these novel transcripts was not performed in the present study, their specific regulatory mechanisms await further experimental confirmation.

### 4.4. Association Between Energy Metabolism and Transcriptional Regulation

As shown in Figure 5A,B, clustering and trend analysis revealed that the “continuously upregulated” and “continuously downregulated” trend subsets dominated among the 618 DEGs. This result indicates that the developmental transition from eggs to larvae in *O. mykiss* is not characterized by random fluctuations but rather by a well-defined programmed temporal regulation. Continuously upregulated genes were mainly enriched in immune and stress-response functions, while continuously downregulated genes were primarily concentrated in amino acid and energy metabolism pathways. This complementary expression pattern reflects the organism’s resource allocation strategy at critical developmental junctures: when yolk reserves are depleted, larvae must redirect limited energy from growth and storage toward fundamental survival activities, including immune defence and stress adaptation [51]. This finding is consistent with the concept of “developmental reprogramming,” whereby organisms achieve rapid physiological state transitions through systemic gene expression adjustments at key life-history stages [52].

The proportion of DEGs identified in this study (618 of 44,665 expressed genes, approximately 1.4%) is within the range reported for comparable developmental transitions in salmonids and other teleosts. For example, transcriptomic comparisons between consecutive developmental stages in Atlantic salmon and rainbow trout typically report DEG proportions of 1–5%, depending on stage interval, sequencing depth, and statistical thresholds. The relatively modest proportion observed here is consistent with the short developmental interval between the eyed-egg stage and 7 days post-hatching, during which transcriptional changes are substantial but not global. The clear separation of the two groups in PCA and the high intra-group reproducibility further support the biological relevance of the identified DEGs.

### 4.5. Limitations

Several limitations of this study should be acknowledged. First, all experimental samples were derived from triploid rainbow trout. Triploidy may impose genomic stress that affects gene expression and developmental physiology. Accordingly, these findings should be interpreted in the context of triploid physiology, and extrapolation to diploid *O. mykiss* requires caution. Second, our analysis was based on only two time points—eyed eggs at 24 dpf and larvae at 31 dpf (approximately 7 dph)—without continuous temporal sampling. The interval between these two points was approximately 7 days (24 dpf to 31 dpf), corresponding to approximately 112 degree-days. Consequently, the dynamic details of gene expression changes during development could not be fully captured, nor could we distinguish between transient responses directly triggered by the hatching event and programmed developmental changes [53]. Third, the causal relationship between differentially expressed genes and phenotypic changes has not been validated through functional experiments such as gene knockout or pharmacological inhibition [54]. Although both DEG-based enrichment and GSEA consistently support the metabolic remodelling during this developmental transition, the number of core differentially expressed genes per pathway remains small (only 2–3 genes per pathway). Meanwhile, GSEA results are inherently limited by the completeness of the reference genome annotation and predefined gene sets. The biological significance of these enrichment findings should therefore be interpreted with caution. Fourth, the prediction of novel transcripts relied on the existing reference genome annotation and was not experimentally validated [55]. Fifth, the samples used in this study were limited to whole embryos/larvae at specific developmental stages and could not reflect organ-specific gene expression patterns [56]. Sixth, the results were obtained under laboratory conditions, and their applicability to natural populations requires further verification [57]. Finally, it should be noted that whole eggs and whole larvae differ not only in developmental stage but also in tissue composition. The newly hatched larvae contain newly formed tissues such as muscle, intestine, and gills, which are absent or rudimentary in eyed eggs. Therefore, part of the observed transcriptional differences may reflect the emergence of new cell types rather than developmental reprogramming per se. Future studies using tissue-specific transcriptomics or single-cell approaches will be needed to disentangle these contributions.

## 5. Conclusions

In summary, this study systematically revealed transcriptomic reprogramming features accompanying the developmental transition from eggs to newly hatched larvae in *O. mykiss* through RNA-seq analysis. Differentially expressed genes were primarily associated with two core modules: remodelling of immune-related gene expression and metabolic pathway changes. KEGG enrichment analysis identified only Glycolysis/Gluconeogenesis as significantly enriched, whereas nitrogen metabolism and tryptophan metabolism showed enrichment trends without statistical significance after multiple-testing correction. Among the immune system process DEGs, 4 were upregulated and 11 were downregulated, indicating complex remodelling rather than simple activation. Because these metabolic pathways contained only a small number of DEGs and showed mixed regulation, no overall pathway-level direction was inferred. These findings suggest that the developmental transition may involve a shift from anabolic storage to catabolic utilization, together with complex regulation of immune-related gene expression. The discovery of putative novel transcripts further enriches the genome annotation of *O. mykiss* and provides candidate molecular resources for future developmental biology research. Although causal relationships require experimental validation, this study provides useful clues for understanding the molecular basis of early survival strategies in teleosts. Future research should integrate temporal sampling, tissue-specific transcriptomics, and functional validation to further dissect the key gene networks regulating early development in *O. mykiss*. This study provides three valuable contributions to existing salmonid developmental knowledge. First, genome-wide transcriptome profiles of rainbow trout at eyed-egg and newly hatched larval stages are established as baseline resources for studying this commercially important fish. Second, key differentially expressed genes and functional pathways driving immune-related remodelling and metabolic reprogramming during hatching are systematically characterized, revealing coordinated patterns between these two core biological processes. Third, the complete list of differentially expressed genes provided in Supplementary Materials offers reference data for future comparative and functional developmental studies across salmonids.

## Figures and Tables

**Table 1:** Alignment statistics.

Sample	Total Reads	Total Mapped	Multiple Mapped	Uniquely Mapped
A_1	46,361,054	44,177,164 (95.29%)	3,631,336 (7.83%)	40,545,828 (87.46%)
A_2	37,941,688	36,260,808 (95.57%)	3,154,816 (8.31%)	33,105,992 (87.25%)
A_3	43,513,158	41,324,615 (94.97%)	3,334,513 (7.66%)	37,990,102 (87.31%)
B_1	42,231,640	40,125,666 (95.01%)	3,164,350 (7.49%)	36,961,316 (87.52%)
B_2	40,244,366	38,376,132 (95.36%)	3,098,095 (7.70%)	35,278,037 (87.66%)
B_3	38,796,990	36,866,615 (95.02%)	2,903,355 (7.48%)	33,963,260 (87.54%)

**Table 2:** Novel transcript classification.

Class Code	Description	Number
=	Complete match of intron chain	134,606
c	Contained within known transcript	5
i	Entirely within reference intron	654
j	Potential novel isoform (at least one splice junction matches reference)	134,770
o	Exonic overlap with reference transcript	1170
u	Unknown, intergenic transcript (new gene)	2902
x	Exonic overlap on opposite strand	1186

**Table 3:** Functional annotation statistics of expressed and reference-annotated genes and transcripts in *O. mykiss*.

	Genes Expressed in Samples (%)	Transcripts Expressed in Samples (%)	Reference-Annotated Genes (%)	Reference-Annotated Transcripts (%)
GO	29,550 (66.16)	53,233 (57.43)	43,119 (59.25)	73,025 (54.25)
KEGG	33,428 (74.84)	73,342 (79.12)	46,135 (63.40)	96,408 (71.62)
EggNOG	39,834 (89.18)	85,004 (91.70)	42,819 (58.84)	99,961 (74.26)
NR	43,576 (97.56)	91,118 (98.30)	65,718 (90.31)	126,330 (93.85)
Swiss-Prot	38,958 (87.22)	83,506 (90.09)	41,230 (56.66)	97,523 (72.45)
Pfam	37,867 (84.78)	79,911 (86.21)	39,042 (53.65)	91,837 (68.22)
Total_anno	43,593 (97.60)	91,167 (98.35)	65,940 (90.61)	126,601 (94.05)
Total	44,665 (100.00)	92,695 (100.00)	72,773 (100.00)	134,611 (100.00)

**Table 4:** Differential expression summary.

Diff Group	Total DEG	Up	Down
B_vs._A	618	297	321

**Table 5:** Selected GO enrichment results.

GO ID	Description	Term Type	B_vs._A_G Num	B_vs._A_G_Up Num	B_vs._A_G_Down Num
GO:0002376	immune system process	BP	15	4	11
GO:0065007	biological regulation	BP	125	72	53
GO:0042592	homeostatic process	BP	9	4	5
GO:0022414	reproductive process	BP	3	1	2
GO:0009987	cellular process	BP	208	97	111
GO:0032502	developmental process	BP	33	20	13
GO:0032501	multicellular organismal process	BP	26	19	7
GO:0051179	localization	BP	35	20	15
GO:0050896	response to stimulus	BP	43	18	25

**Table 6:** Selected KEGG enrichment results of DEGs.

Pathway ID	Description	Rich Factor	*p* Value	padjust
map00910	Nitrogen metabolism	0.027027	0.390471	1
map00010	Glycolysis/Gluconeogenesis	0.066176	8.9969732269861 × 10^−5^	0.009717
map00380	Tryptophan metabolism	0.012987	0.643311	1

## Data Availability

The raw RNA sequencing data generated in this study have been deposited in NCBI under BioProject accession number PRJNA1477098.

## Supplementary Materials

The following supporting information can be downloaded at https://www.mdpi.com/article/10.3390/biology15201872/s1, Table S1: Complete dataset of differentially expressed genes (DEGs) between the eyed-egg stage and the larval stage, including gene identifiers, gene names, functional annotations, fold change values, *p*-values and adjusted *p*-values. Table S2: Full statistical results of Gene Ontology (GO) gene set enrichment analysis (GSEA). Table S3: Full statistical results of Kyoto Encyclopedia of Genes and Genomes (KEGG) pathway enrichment analysis (GSEA). Figure S1: Bubble plot of GO gene set GSEA enrichment results. Dot colour represents the adjusted *p*-value (Padjust), dot size indicates the number of genes contained in each gene set, and the *x*-axis shows the normalized enrichment score (NES). Figure S2: Bubble plot of KEGG pathway GSEA enrichment results. Dot colour represents the adjusted *p*-value (Padjust), dot size indicates the number of genes contained in each pathway, and the *x*-axis shows the normalized enrichment score (NES). Figure S3: Enrichment plot of the “structural constituent of eye lens” gene set (GO:0005212). Figure S4: Enrichment plot of the “glycolysis-gluconeogenesis” pathway (map00010).

## References

[1] Holler K., Neuschulz A., Drewe-Boss P., Mintcheva J., Spanjaard B., Arsie R., Ohler U., Landthaler M., Junker J.P. (2021). Spatio-temporal mRNA tracking in the early zebrafish embryo. Nat. Commun..

[2] Majewski M., Kasica N., Jakimiuk A., Podlasz P. (2018). Toxicity and cardiac effects of acute exposure to tryptophan metabolites on the kynurenine pathway in early developing zebrafish (*Danio rerio*) embryos. Toxicol. Appl. Pharmacol..

[3] Aramburu O., Pardo B.G., Jimenez-Gonzalez A., Blanco-Hortas A., Macqueen D., Bouza C., Martinez P. (2025). Epigenomics of embryogenesis in turbot. Genome Res..

[4] Aramburu O., Pardo B.G., Jimenez-Gonzalez A., Blanco-Hortas A., Macqueen D.J., Bouza C., Martínez P. (2024). Epigenomics of embryogenesis in turbot (*Scophthalmus maximus*). bioRxiv.

[5] Guerrero-Peña L., Suarez-Bregua P., Sánchez-Baizán N., Piferrer F., Tena J.J., Rotllant J. (2025). Epigenetic Regulation During Flatfish Metamorphosis: Integrative Omics Analysis of DNA Methylation and Gene Expression in Turbot Brain. bioRxiv.

[6] Li C., Qin C., Chen X., Huang X., Xu S., Yan X., Hu J., Wang Y. (2026). Comparative analysis of muscle structure and weighted gene co-expression network analysis reveals growth mechanisms in fast-growing and slow-growing silver pomfret (*Pampus argenteus*). J. Fish. Biol..

[7] Wu Y., Li Y., Xia W., Chen T., Qiu Y., Chen D., Wang Y., Yan X., Zhang M., Hu J. (2026). Cold and Rewarming Alter Polyunsaturated Fatty Acid Profiles in Silver Pomfret via Regulation of Elongation of Very Long-Chain Fatty Acids (Elovl). J. Agric. Food Chem..

[8] Zhang M., Zhang L., Yuan Z., Xu S., Chen Y., Xu F., Qiu Y., Tang M., Dai Q., Li Y. (2026). Cold Stress Induces Tissue-Specific Lipid Metabolic Responses and Scd1-Mediated Hepatic Apoptosis in Silver Pomfret. Animals.

[9] Zhao L., He K., Xiao Q., Liu Q., Luo W., Luo J., Fu H., Li J., Wu X., Du J. (2021). Comparative transcriptome profiles of large and small bodied large-scale loaches cultivated in paddy fields. Sci. Rep..

[10] Wang Z.R., Li S.Y., Zhang Y.Z., Li Y.A., Huo H.H., Yu C.Q., Zhou Q.B. (2023). Metabolomic and transcriptomic profiling reveals the effect of dietary protein and lipid levels on growth performance in loach (*Paramisgurnus dabryanus*). Front. Immunol..

[11] Tyler C.R., Jobling S., Sumpter J.P. (1998). Endocrine disruption in wildlife: A critical review of the evidence. Crit. Rev. Toxicol..

[12] McKinney G.J., Hale M.C., Goetz G., Gribskov M., Thrower F.P., Nichols K.M. (2015). Ontogenetic changes in embryonic and brain gene expression in progeny produced from migratory and resident *Oncorhynchus mykiss*. Mol. Ecol..

[13] Turner L. (2025). Transcriptomic and Physiological Responses to Abiotic Stressors in Rainbow Trout (*Oncorhynchus mykiss*). Ph.D. Thesis.

[14] Airaksinen S., Rabergh C.M., Sistonen L., Nikinmaa M. (1998). Effects of heat shock and hypoxia on protein synthesis in rainbow trout (*Oncorhynchus mykiss*) cells. J. Exp. Biol..

[15] Bari S.M., Fuad M., Hossain M.J., Ahammad I., Begum M., Helal M.M.U. (2025). Transcriptomic Insights into Heat and Hypoxia Stress in Rainbow Trout: A Meta-Analysis of Public RNA-Seq Data. BMC Genom..

[16] Dubey M.K., Kamalam B.S., Rajesh M., Sarma D., Pandey A., Baral P., Sharma P. (2023). Exposure to different temperature regimes at early life stages affects hatching, developmental morphology, larval growth, and muscle cellularity in rainbow trout, *Oncorhynchus mykiss*. Fish. Physiol. Biochem..

[17] Debernardis R., Palińska-Żarska K., Judycka S., Panda A., Jarmołowicz S., Jastrzębski J.P., de Almeida T.R., Błażejewski M., Hliwa P., Krejszeff S. (2025). Does transcriptome of freshly hatched fish larvae describe past or predict future developmental trajectory?. Aquaculture.

[18] Li B., Dewey C.N. (2011). RSEM: Accurate transcript quantification from RNA-Seq data with or without a reference genome. BMC Bioinform..

[19] Steyn G., Van Vuren J., Grobler E. (1989). A new sperm diluent for the artificial insemination of rainbow trout (*Salmo gairdneri*). Aquaculture.

[20] Iunes R.S. (2022). Neutrophils of diploid and triploid rainbow trouts assessed by flow cytometry. Multidiscip. Sci. J..

[21] Gabillard J.C., Yao K., Vandeputte M., Gutierrez J., Le Bail P.Y. (2006). Differential expression of two GH receptor mRNAs following temperature change in rainbow trout (*Oncorhynchus mykiss*). J. Endocrinol..

[22] Fahliani Z.G., Falahatkar B., Rahdari A. (2025). Effect of light conditions during the incubation period on eggs of rainbow trout brooders fed with different levels of dietary ascorbic acid. J. Fish..

[23] Sempeski P., Gaudin P. (1996). Size-related shift in feeding strategy and prey-size selection in young grayling (*Thymallus thymallus*). Can. J. Zool..

[24] SEQC/MAQC-III Consortium (2014). A comprehensive assessment of RNA-seq accuracy, reproducibility and information content by the Sequencing Quality Control Consortium. Nat. Biotechnol..

[25] Modi A., Vai S., Caramelli D., Lari M. (2021). The Illumina sequencing protocol and the NovaSeq 6000 system. Bacterial Pangenomics: Methods and Protocols.

[26] Hao Z., Liang X., Li G. (2022). Quality control and preprocessing of sequencing reads. Bio-101.

[27] Ngwa J., Wojciechowski R., Zack D.J., Beaty T., Ruczinski I. (2017). Differential Expression Analysis of Gene and Transcript Abundance for Single Cell RNA-Seq Data using STAR and HISAT Aligners. Investig. Ophthalmol..

[28] Pertea M., Pertea G.M., Antonescu C.M., Chang T.C., Mendell J.T., Salzberg S.L. (2015). StringTie enables improved reconstruction of a transcriptome from RNA-seq reads. Nat. Biotechnol..

[29] Shumate A., Wong B., Pertea G., Pertea M. (2022). Improved transcriptome assembly using a hybrid of long and short reads with StringTie. PLoS Comput. Biol..

[30] The Gene Ontology Consortium (2001). Creating the gene ontology resource: Design and implementation. Genome Res..

[31] Kanehisa M., Goto S. (2000). KEGG: Kyoto encyclopedia of genes and genomes. Nucleic Acids Res..

[32] Buchfink B.J., Barbe E., Ashkenazy H., Reuter K., Kennedy J.A., Drost H.G. (2026). Clustering the protein universe of life using DIAMOND DeepClust. Nat. Methods.

[33] Zhang C., Zhang B., Vincent M., Zhao S. (2016). Bioinformatics tools for RNA-seq gene and isoform quantification. Next Gener. Seq. Appl..

[34] Dos Santos K.C.G., Desgagne-Penix I., Germain H. (2020). Custom selected reference genes outperform pre-defined reference genes in transcriptomic analysis. BMC Genom..

[35] Venables W.N., Smith D.M., The R Core Team (2009). An Introduction to R.

[36] Wen G. A simple process of RNA-sequence analyses by Hisat2, Htseq and DESeq2. Proceedings of the 2017 International Conference on Biomedical engineering and Bioinformatics.

[37] Benjamini Y., Hochberg Y. (1995). Controlling the false discovery rate: A practical and powerful approach to multiple testing. J. R. Stat. Soc. Ser. B.

[38] Lima D.A., Goncalves L.O., Reis-Cunha J.L., Guimaraes P.A.S., Ruiz J.C., Liarte D.B., Murta S.M.F. (2023). Transcriptomic analysis of benznidazole-resistant and susceptible *Trypanosoma cruzi* populations. Parasit. Vectors.

[39] Yuan B., Chen Y., Wu Z., Zhang L., Zhuang Y., Zhao X., Niu H., Cheng J.C.-H., Zeng Z. (2018). Proteomic profiling of human hepatic stellate cell line LX2 responses to irradiation and TGF-β1. J. Proteome Res..

[40] Trede N.S., Zon L.I. (1998). Development of T-cells during fish embryogenesis. Dev. Comp. Immunol..

[41] Takemura A. (1993). Changes in an immunoglobulin M (IgM)-like protein during larval stages in tilapia, *Oreochromis mossambicus*. Aquaculture.

[42] Martínez G., Peña E., Martínez R., Camarillo S., Burggren W., Álvarez A. (2021). Survival, growth, and development in the early stages of the tropical gar *Atractosteus tropicus*: Developmental critical windows and the influence of temperature, salinity, and oxygen availability. Fishes.

[43] Zhou Z.Y., Jian Z.R., Gu J.L., Jiang X.Y., He J., Gao Y.C., Lian S., Bai S.J., Lu C.W., Kuang L.F. (2026). Single-cell sequencing reveals metabolic reprogramming and immune regulation underlie the maintenance of teleost granulomas. Fish. Shellfish Immunol..

[44] Özcan F., Arserim N.B. (2026). Antibacterial immunity in teleost fish: Integrating innate and adaptive responses for sustainable aquaculture. Vet. Immunol. Immunopathol..

[45] Conceição L.E., Morais S., Rønnestad I. (2007). Tracers in fish larvae nutrition: A review of methods and applications. Aquaculture.

[46] Jiang Y., Ding Z., Hu R., Magnuson J.T., Li S., Wang D., Zhou S., Guo Y., Wang Y., Liu Y. (2026). PPARα Antagonism Rescues Chlorpyrifos-Induced Neuro-Visual Toxicity in Zebrafish (*Danio rerio*) Larvae. Toxics.

[47] Hoseini S.M., Pérez-Jiménez A., Costas B., Azeredo R., Gesto M. (2019). Physiological roles of tryptophan in teleosts: Current knowledge and perspectives for future studies. Rev. Aquac..

[48] Aniseia Y., Waltari E., Huang H., Lima L., Rahman G., Frank M., Zhou A., Kim Y.-J., Paras J., Baker S. (2026). RNAquarium: An archive-scale atlas of zebrafish gene expression coupled with pan-taxonomic profiling reveals diverse viral drivers of transcriptomic states. bioRxiv.

[49] Pandey S., Moyer A.J., Thyme S.B. (2023). A single-cell transcriptome atlas of the maturing zebrafish telencephalon. Genome Res..

[50] ÇİLTAŞ A., Al-Soughaier E.S. (2025). Comprehensive identification and functional characterization of non-coding RNAS in fish: Regulatory mechanisms and bioinformatics approaches. Preprint.

[51] Hunt von Herbing I. (2024). Energetic Costs of Stress in Developing Fishes: Quantifying Allostasis and Allostatic Load. Integr. Comp. Biol..

[52] Louro B., Marques J.P., Manchado M., Power D.M., Campinho M.A. (2020). Sole head transcriptomics reveals a coordinated developmental program during metamorphosis. Genomics.

[53] Liu M., Wang M., Jin C., Li J., Zheng D., Tang H., Zhai Z., Bao Z., Wang B., Hu J. (2026). Integrative analysis of transcriptome dynamics and Notch signaling pathways reveals molecular basis of egg quality in *Plectropomus leopardus*. Aquaculture.

[54] Hoodless L.J., Lucas C.D., Duffin R., Denvir M.A., Haslett C., Tucker C.S., Rossi A.G. (2016). Genetic and pharmacological inhibition of CDK9 drives neutrophil apoptosis to resolve inflammation in zebrafish in vivo. Sci. Rep..

[55] Fan X., Zhang X., Wu X., Guo H., Hu Y., Tang F., Huang Y. (2015). Single-cell RNA-seq transcriptome analysis of linear and circular RNAs in mouse preimplantation embryos. Genome Biol..

[56] Sveen L.R., Robinson N., Krasnov A., Daniels R.R., Vaadal M., Karlsen C., Ytteborg E., Robledo D., Salisbury S., Dagnachew B. (2023). Transcriptomic landscape of Atlantic salmon (*Salmo salar* L.) skin. G3 Genes Genomes Genet..

[57] Alvarez M., Schrey A.W., Richards C.L. (2015). Ten years of transcriptomics in wild populations: What have we learned about their ecology and evolution?. Mol. Ecol..

